# Quantitative Determination of Regional Lesion Volume and Distribution in Children and Adults with Relapsing-Remitting Multiple Sclerosis

**DOI:** 10.1371/journal.pone.0085741

**Published:** 2014-02-26

**Authors:** Rezwan Ghassemi, Sridar Narayanan, Brenda Banwell, John G. Sled, Manohar Shroff, Douglas L. Arnold, on behalf of the Canadian Pediatric Demyelinating Disease Network

**Affiliations:** 1 Montreal Neurological Institute, McGill University, Montreal, Quebec, Canada; 2 The Hospital for Sick Children, University of Toronto, Toronto, Ontario, Canada; 3 The Children's Hospital of Philadelphia, Philadelphia, Pennsylvania, United States of America; University of Minnesota, United States of America

## Abstract

**Introduction:**

Onset of MS occurs during childhood in about 5% of cases. It is unclear whether very young age at MS onset, when the nervous system is still myelinating, affects MS lesion accrual or regional distribution.

**Objective:**

To compare the frequency, volume and distribution of T2 and T1 lesions in children and adults with relapsing-remitting multiple sclerosis (RRMS).

**Methods:**

Lesions were segmented on T2- and T1-weighted MRI images from 29 children and 29 adults with RRMS, matched for disease duration.

**Results:**

All subjects exhibited T2-weighted brain lesions. Children had higher whole-brain T2-weighted-lesion-volume (T2LV) compared to adults (mean (SD) in cm^3^: 12.76(2.7) vs. 10.03(3.4), p<0.0013). The supratentorial-T2LV was similar in children and adults (8.45(1.7) vs. 7.94(1.7), mean (SD), p = 0.2582), but adults were more likely to have supratentorial lesions (96.5% vs. 68.9%, p<0.012). Children were more likely to have infratentorial-T2-weighted lesions (75.9% vs. 43.4%, p<0.03), specifically in the brainstem (62.1% vs. 26.7%, p<0.019) and the pons (48.3% vs. 17.24%, p<0.024), had higher infratentorial-T2-weighted-lesion counts (4.1(5.6) vs. 1.45(2.3), p<0.021), a greater infratentorial-T2LV (4.31(2.7) vs. 2.08(2.4), p<0.0013), and a greater infratentorial-T1-weighted-lesion-volume (T1LV) (3.7(2.5) vs. 1.08(1.9), p<0.0007). Whole-brain-T1LV was higher in children (9.3(2.5) vs. 6.43(2.1), p>0.001). Adult MS patients had higher supratentorial-T1LV (5.5(0.92) vs. 6.41(2.1), mean (SD), p<0.034), whereas children were more likely to have infratentorial-T1-weighted lesions (58.6% vs. 23.3%, p<0.015).

**Discussion:**

Onset of MS during childhood is associated with a higher volume of brain lesions in the first few years of disease relative to adults. Children with MS are more likely than adults to have T2 and T1 lesions in the infratentorial white matter, raising the possibility of preferential immune targeting of more mature myelin. Children with MS have a lower supratentorial T1 lesion burden, possibly reflecting more effective remyelination and repair in brain regions that are still engaged in active primary myelination.

## Introduction

The onset of MS during childhood and adolescence occurs in the context of ongoing maturation of neural networks and during primary myelination. The very young age of pediatric MS patients also inherently limits the potential for a prolonged subclinical phase of the disease.

MRI provides a window into the inflammatory aspects of MS, as illustrated by T2-weighted lesion analyses, and to the relative severity of tissue injury as demonstrated by T1-weighted focal lesions and changes in brain volume [1]. While the correlation of focal white matter lesions with clinical disability may be modest in clinical trials, where the range of disease is limited, over the entire spectrum of disease severity the correlation of total focal white matter lesion volume and clinical disability is relatively strong [2].

The onset of MS during childhood is being increasingly recognized. Pediatric MS is characterized by a relapsing-remitting disease course, and with a relatively low risk of early physical disability [3]. Recent studies have reported the key MRI features that characterize MS in children [4]–[6], and have supported the applicability of the 2010 McDonald diagnostic criteria for MS [7] in the pediatric population (with some specific considerations). More direct comparison of lesion distribution and volume between pediatric and adult-onset MS cohorts, however, are rare [5]. We previously evaluated lesion characteristics of children with a first attack of demyelination to the lesion distribution and volumes of an adult MS cohort [8]. In the present work, we evaluate lesion count, distribution and volume in pediatric and adult MS patients matched for disease duration (time since first attack) in order to explore whether the low risk of early disability in children relates to a lower lesion burden, and to evaluate whether lesion distribution in children is influenced by the potential for greater lesion repair in the context of active, primary, age-expected myelination. To this end, we used advanced image processing techniques to segment tissues in brain images, compute regional lesion load, and generate lesion frequency maps for children and adults with RRMS.

## Materials and Methods

### Demographics

For the adult patients written informed consent was obtained from all adult-onset MS patients. The consent form and study were approved by the Research Ethics Boards at the Montreal Neurological Institute and Hospital. For the pediatric patients written informed consent was obtained from the legal guardians of all pediatric-onset MS patients enrolled prior to age 18 years. Assent was obtained verbally and when possible, in writing, from all pediatric patients old enough to fully comprehend the assent document. The study was approved by the Research Ethics Boards at the Hospital for Sick Children.

Participants included 29 children with RRMS (mean age at time of scan (SD): 15.33 (2.1) yrs, age range: 10.08–17.82 yrs; 23 female and 6 male, mean age at time of first attack: 11.8 (3.64) yrs, age range: 4.7–16.8 yrs) (Table 1). Cognitive and neuroimaging features of some of these pediatric MS patients have been included in our prior work [9]. A comparison group of 29 adult patients with RRMS was selected retrospectively from a prior imaging study (mean age at time of scan (SD): 32.53 (6.9) yrs, range: 20.61–48.62; 21 female and 8 male, mean age at time of first attack: 29.4 (7.4) yrs, range: 18.4–46.44). Pediatric and adult-onset MS groups were matched for disease duration (defined as the time from first clinical demyelinating attack to the date of MRI) and sex. RRMS in the children was defined by the occurrence of two or more demyelinating attacks (separated by more than 30 days and involving multiple areas of the CNS, or by MRI evidence of new lesions over time [10]. The 2010 McDonald criteria were not validated for use in children at the time the present cohort was selected, and thus none of the children were diagnosed with MS on the basis solely of a first demyelinating attack and concurrent presence of MRI features meeting these 2010 criteria at onset [7]. Children with an initial attack meeting criteria for acute disseminated encephalomyelitis (ADEM) were required to have two or more non-ADEM attacks for MS diagnosis. RRMS in the adults was defined by Poser criteria [11].

**Table 1:** Demographic data in the pediatric and adult groups.

Data	Pediatric MS	Adult MS
Number of patients (N)	29	29
Sex (F/M, % female)	23/6 (76.6% female)	21/8 (72.4% female)
Age at time of first attack, years Mean (SD)	11.8 (3.64)	29.4 (7.4)
Age at time of first attack, years (Range)	4.7–16.8	18.4–46.44
Disease duration, years Mean (SD)	4.2 (2.6)	4 (2.9)
Disease duration, years (Range)	0.15–10.36	0.24–9.54
Age at time of scan, years Mean (SD)	15.33 (2.1)	32.53 (6.9)
Age at time of scan, years (Range)	10.08–17.82	20.61–48.62

The pediatric and adult MS groups were matched for disease duration (t-test, p = 0.78) and proportion of females (Fisher's exact test, p = 0.76).

### MRI acquisition

Children with MS were scanned on a single 1.5T GE TwinSpeed Excite 12.0 scanner at the Hospital for Sick Children. The standardized MRI protocol included an axial dual-contrast PD-weighted/T2-weighted fast spin-echo sequence acquired parallel to the line connecting the inferior aspects of the genu and splenium of the corpus callosum (callosal line) (TR/TE1/TE2 = 3500/15/63 ms, 256×256 matrix, 1 signal average, 250 mm FOV, 2 mm thick contiguous slices, 90° flip angle), and a sagittal T1-weighted 3D spoiled gradient-recalled echo sequence (TR/TE = 22/8 ms, 1 signal average, 250 mm FOV, 1.5 mm slice thickness, 30° flip angle).

MRI examinations of the adult MS group were performed using a 1.5T Philips Gyroscan ACS II (Philips Medical Systems, Best, The Netherlands) at the Montreal Neurological Institute. Proton density and T2-weighted images were obtained using an axial dual-contrast PD-weighted/T2-weighted fast spin-echo sequence (TR/TE1/TE2 = 2075/32/90, 256×256 matrix, 1 signal average, 250 mm FOV, 3 mm thick contiguous slices, 90° flip angle), followed by an axial T1-weighted 3D spoiled gradient-recalled echo sequence (TR/TE = 35/10 ms, 256×256 matrix, 1 signal average, 3 mm slice thickness, 40° flip angle). Both sequences were angulated parallel to the callosal line.

### Data analysis

All images were evaluated for adequate signal-to-noise ratio, freedom from significant motion or other artifact, and consistency of the sequence parameters [12]. To account for differing slice thickness between images from children and adults, PD-, T2- and T1-weighted images from the children were resampled to match the sampling of the images from adults, i.e. with axial slices of 3 mm thickness and in-plane voxel dimensions of 0.98 mm×0.98 mm. A pre-processing routine was run on all images to correct for intensity non-uniformity [13], to remove skull and scalp, and to linearly register the PD- and T1-weighted images to the T2-weighted image in order to provide voxel-wise anatomical alignment across the modalities [14]. The intensity range was normalized within each image, using a two-piece linear transformation similar to the one described by Nyul et al. [15]

### Lesion segmentation

Supratentorially, T2-weighted (T2w) lesion segmentation was performed in a semi-automated manner. An initial segmentation was performed using a locally-developed, automated, multi-spectral Bayesian technique [16], as previously described [8]. Using an interactive, mouse-driven visualization software package (DISPLAY, McConnell Brain Imaging Centre, MNI), the T2-weighted lesion labels output by the initial segmentation were superimposed on the T1-, T2-, and PD-weighted images, carefully reviewed and, if necessary, manually corrected. Due to different MRI intensity characteristics between supra- and infratentorial lesions, infratentorial T2-weighted lesions were not reliably identified using the automated methods and were segmented manually.

T1-weighted (T1w) hypointense regions first were confirmed to be bright on T2-weighted images. T1 hypointensities then were segmented automatically by applying an intensity threshold relative to the mean intensity of surrounding normal-appearing white matter. As the pediatric and adult patients were scanned using slightly different protocols, the contrast of the T1-weighted sequences differed between the two groups. To minimize any potential bias in the T1-weighted lesion borders that could occur as a consequence of protocol differences, we used existing images from a separate group of 10 adult MS patients scanned using both the pediatric and adult protocols (on the MNI Philips scanner) to determine the intensity thresholds that yielded equivalent T1-weighted lesion volumes on the same individuals for both protocols. Based on these results, a T1-weighted intensity threshold of 85% of the mean intensity of surrounding white matter for the pediatric protocol was determined to be equivalent to a threshold of 83% for the adult protocol.

To enable spatial analyses over multiple subjects and to correct for differences in head size, the T2-weighted and T1-weighted images and lesion labels for each subject were transformed into stereotaxic space based on a non-linear automated multiscale feature-matching algorithm [17].

For each patient group (pediatric and adult), two lesion frequency masks were calculated in stereotaxic space for T2-weighted lesions; one for the supratentorial region, and one for the infratentorial region. An average T2-weighted anatomical image also was created for each group.

The total number of T2w and T1w lesions, and the total number of lesions in the supra- and infratentorial compartments, were calculated for each patient in native space. The minimum size criterion for counting a T2-weighted lesion was 3 voxels (9 mm^3^). Clusters of lesion voxels were considered as distinct lesions only if they were separated by at least one voxel.

The proportion of pediatric and adult RRMS patients with lesions in the supra- and infratentorial space, and then more specifically in the cerebellum and the brainstem as a whole, as well as for the midbrain, pons and medulla oblongata, were assessed for each group. The cerebellar peduncles were considered to be part of the brainstem.

### Statistical analysis

T2- and T1-weighted lesion volumes were calculated separately for the supratentorial and infratentorial regions in each group. Because of skew in the distribution of lesion volumes, a logarithmic transformation (log (variable+constant)) was applied to better approximate a normal distribution and allow the use of parametric statistical tests [18]. Continuous non-normalized variables were analyzed using the non-parametric Mann-Whitney U-test. Student's t-tests were used to assess whether normalized lesion volumes differed significantly between the pediatric and adult groups.

Fisher's exact test was used to assess whether significant differences existed between the pediatric MS and adult MS groups in terms of the incidence of T2 and T1-weighted lesions in the supratentorial and infratentorial regions.

For patients exhibiting infratentorial T2-weighted lesions, Fisher's exact test also was applied to test for significant differences between the two groups in the proportion of T2-weighted lesions located within the brainstem and each subregion (midbrain, pons and medulla oblongata).

After defining a periventricular regional mask, as previously described [19], lesion volumes in the non-periventricular versus periventricular brain regions were compared between the pediatric and adult MS groups using the Mann-Whitney U test.

T2-weighted lesion counts were performed separately for the supratentorial and infratentorial regions and the entire brain in each group. T-tests were used to assess whether lesion counts were significantly different between pediatric and adult MS groups.

Given that our comparisons were pre-specified, guided by prior studies, we considered a p-value of 0.05 as the threshold for significance.

## Results

Children and adults with MS had similar total brain T2-weighted lesion counts (35.9 (40.8) vs. 28.8 (22.01), p = 0.41), but the total T2-weighted lesion volume in the whole brain was higher in the children compared to the adults; (12.76 (2.7) cm^3^ vs. 10.03 (3.4) cm^3^, p<0.0013).

**Figure 1:**
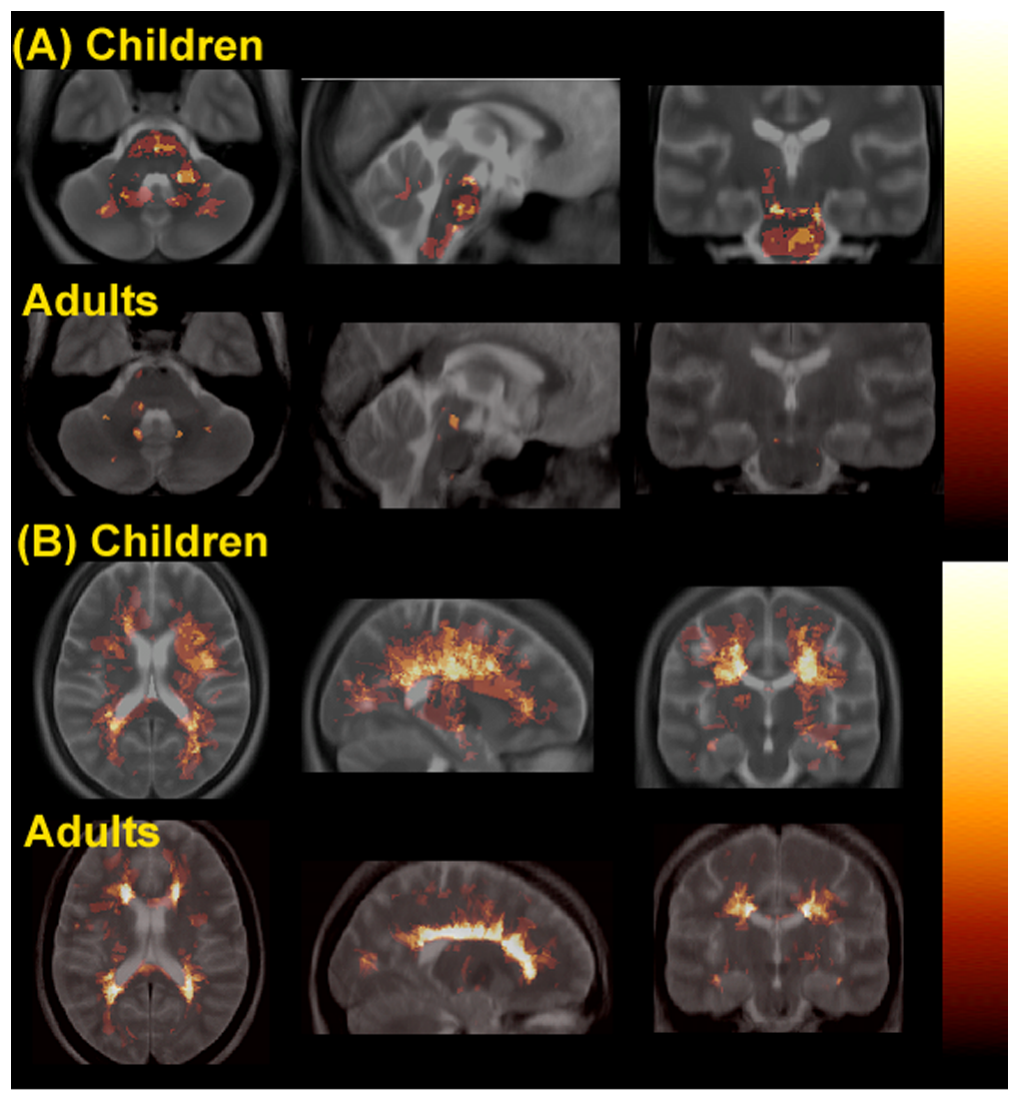
Lesion frequency maps (axial (left), sagittal (middle) and coronal (right) views) in the hot metal color scale showing the infratentorial (A) and supratentorial (B) T2-weighted lesion distributions in children with RRMS (rows 1 and 3) vs. adults with RRMS (rows 2 and 4), superimposed on the average T2-weighted structural images (in grey scale) for each group. The intensity of each voxel in the overlay represents the group frequency for lesions at each location.

Evaluating supra- and infratentorial regions separately revealed regional differences. In the supratentorial region, the number (31.8 (38.44) vs. 27.3 (20.91), p>0.58) and volume (8.45 (1.7) cm^3^ vs. 7.94 (1.7) cm^3^, mean (SD), p>0.26) of T2w lesions were comparable between children and adults with RRMS (Table 2). In the infratentorial region, children with MS had a higher incidence of T2-weighted lesions than adults (N: 22 (75.9%) vs. 13 (43.4%), p<0.031), specifically in the brainstem as a whole (18 (62.1%) vs. 8 (26.7%), p<0.019) and the pons in particular (14 (48.3%) vs. 5 (17.2%), p<0.024) (Figure 1 and Table 2). Lesions were infrequent in the midbrain or medulla in both children and adults, which precluded comparison of lesion distribution in these regions. When evaluating the infratentorial regions as a whole, the number of infratentorial T2-weighted lesions was significantly higher in children with RRMS compared to adults (4.1 (5.6) vs. 1.45 (2.3), p<0.021), and children exhibited greater infratentorial T2-weighted lesion volume (4.31 (2.7) cm^3^ vs. 2.08 (2.4) cm^3^, p<0.0013).

**Table 2:** Supratentorial and infratentorial incidence and volume of T2 and T1-weighted lesions.

Variable	Statistic	Pediatric MS	Adult MS	*P* value
Number of patients	N	29	29	-
Number of patients exhibiting supratentorial T2w lesions	N (%)	29 (100%)	29 (100%)	p = 1.000^1^
Number of patients exhibiting supratentorial T1w lesions	N (%)	20 (68.9%)	28 (96.5%)	p<0.012^1^
Number of patients exhibiting infratentorial T2w lesions	N (%)	22 (75.9%)	13 (43.4%)	p<0.031^1^
Number of patients exhibiting infratentorial T1w lesions	N (%)	17 (58.6%)	7 (23.3%)	p<0.015^1^
Number of patients exhibiting brainstem T2w lesions	N (%)	18 (62.1%)	8 (26.7%)	p<0.019^1^
Number of patients exhibiting pontine T2w lesions	N (%)	14 (48.3%)	5 (17.24%)	p<0.024^1^
Total T2w lesion volume in entire brain, cm^3^	Mean (SD)	12.6 (2.7)	10.03 (3.4)	p<0.001^2^
Total T1w lesion volume in entire brain, cm^3^	Mean (SD)	9.3 (2.5)	6.43 (2.1)	p<0.001^2^
T2w supratentorial lesion volume, cm^3^	Mean (SD)	8.45 (1.7)	7.94 (1.7)	p = 0.26^2^
T1w supratentorial lesion volume, cm^3^	Mean (SD)	5.5 (0.92)	6.41 (2.1)	p<0.037^2^
T2w infratentorial lesion volume, cm^3^	Mean (SD)	4.3 (2.7)	2.08 (2.4)	p<0.0013^3^
T1w infratentorial lesion volume, cm^3^	Mean (SD)	3.7 (2.5)	1.08 (1.9)	p<0.0007^3^
Ratio of T1∶T2-weighted supratentorial lesion volumes	Mean (SD)	0.67 (0.15)	0.80 (0.2)	p<0.007
Ratio of T1∶T2-weighted infratentorial lesion volumes	Mean (SD)	0.81 (0.53)	0.52 (0.7)	p = 0.081
T2w periventricular lesion volume, cm^3^	Mean (SD)	7.5 (2.2)	7.1 (2.7)	p = 0.9124^2^
T2w non-periventricular lesion volume, cm^3^	Mean (SD)	7.7 (1.8)	6.6 (1.95)	p = 0.067^2^
T2w supratentorial lesion counts	Mean (SD)	31.8 (38.44)	27.3 (20.91)	p = 0.58^2^
T2w infratentorial lesion counts	Mean (SD)	4.1 (5.6)	1.45 (2.3)	p<0.021^2^
Total T2w lesion count in entire brain	Mean (SD)	35.9 (40.8)	28.8 (22.01)	p = 0.41^2^

1 measured using Fisher's exact test;

2 measured using two-tailed t-test,

3 measured using non-parametric Wilcoxon test.

We specifically measured the volume of lesions within the periventricular region mask, given that periventricular lesions are an important component of current diagnostic criteria for MS [20]. Periventricular T2-weighted lesion volumes were nearly identical between the pediatric and adult MS groups (7.5 (2.2) vs. 7.1 (2.7), p>0.9124).

Whole-brain T1-weighted lesion volume was higher in children compared to adults (9.3 (2.5) cm^3^ vs. 6.43 (2.1) cm^3^, p<0.001). However, regionally, adult MS patients were more likely to exhibit supratentorial T1-weighted lesions (28 (96.5%) vs. 20 (68.9%), p<0.01), had greater supratentorial T1-weighted lesion volumes (5.5 (0.92) cm^3^ vs. 6.41 (2.1) cm^3^, mean (SD), p<0.037), and showed higher supratentorial ratios of T1w∶T2w lesion volumes (0.80 (0.2) vs. 0.67 (0.15), p<0.007). Relative to adults, the pediatric MS patients exhibited a greater frequency of infratentorial T1-weighted lesions (17 (58.6%) vs. 7 (23.3%), p<0.015), and a greater infratentorial T1-weighted lesion volume (3.77 (2.5) cm^3^ vs. 1.08 (1.9) cm^3^, p<0.0007). The infratentorial ratio of T1w∶T2w lesion volume was not different between the pediatric and adult MS groups (0.81 (0.53) vs. 0.52 (0.7), p>0.08).

## Discussion

We compared T2 and T1-weighted lesion volume, counts and lesion distribution between children and adults with RRMS matched for disease duration. Despite their young age, children with MS accrued greater overall volumes of T2 and T1-weighted lesion than adult RRMS patients. Regional analyses revealed that the infratentorial region was more affected in children than in adults with MS.

Our findings are in agreement with those of Yeh et al. [5] who reported comparable overall T2w lesion volume between children and adults and a trend towards higher T1w lesion volume in children with RRMS [5]. Regional lesion volumes were not assessed in that report.

Given the time course of normal primary brain myelination, we deliberately sought to compare the more developmentally mature white matter of the brainstem and cerebellum with the actively myelinating supratentorial white matter [21]–[23]. We showed that pediatric RRMS patients were more likely than adult-onset MS patients to have infratentorial lesions, potentially implicating a preferential targeting of more mature white matter in children.

A tendency for large T2 lesions has been reported in pediatric MS [24], [25], and thus we were concerned that the higher T2w lesion volume in the pediatric MS patients might reflect larger lesions, rather than a greater number of discrete lesions. However, total lesion counts did not differ between the pediatric and adult-onset MS groups. The mean lesion count in the current pediatric MS cohort (35.9), is very similar to the total lesion count (43.56) obtained as part of our prior pediatric MS studies, in which we utilized a manual lesion identification technique in a group of children with average age of 11 years [26] - suggesting that the pediatric patients included in the present study do not have unusually high lesion volumes. A higher total T2w lesion count in pediatric compared to adult-onset MS patients was reported by Waubant et al [27]. However, the fact that their two populations were not matched for disease duration limits comparison to our study.

Children with established RRMS had a greater likelihood of having infratentorial T2w lesions relative to adults, particularly in the brainstem, and more specifically in the pons. This is in line with our earlier observation in a different group of children that the presence of brainstem lesions, rather than the broader categorization of “infratentorial”, lesions is more characteristic for MS in children [26].

In addition to higher total T1w lesion volume in pediatric-onset MS, the accrual of tissue injury differed by brain region. In the supratentorial region, a smaller fraction of T2–hyperintense lesion was T1-hypointense in the children with MS than in the adults, whereas in the infratentorial region, the T1∶T2 lesion volume ratio was similar. This finding raises the intriguing possibility that active primary myelination, as would be expected in the supratentorial regions during childhood and adolescence, might serve to more effectively remyelinate lesions in this region, limiting T1w lesion formation.

Although the MRI protocol used for the adult MS patients was not identical to the protocol in place for the pediatric MS study, this should not have had a significant influence on our T2-weighted lesion measurements, since the contrast between grey matter, white matter and lesions was similar across groups (contrast ratios in white matter vs. grey matter in the children vs. the adults was 1.36 (0.08) vs. 1.38 (0.12) (p>0.7)). In addition, to avoid a bias in lesion volume estimates due to the thinner slices of the images acquired in the children, we resampled these images to match the 3 mm axial slice thickness of the adult scans. As described, we also performed specific analyses and made minor adjustments to lesion thresholds to control for the small contrast differences in the T1w images obtained with the pediatric and adult MRI protocols. .

Current and emerging new MS therapies are known to reduce new white matter lesion formation [28]–[32]. Our findings, and those of Yeh et al. [5] confirm that pediatric-onset MS is associated with a MRI burden of disease at least as great as is seen in adults with MS - providing compelling rationale to ensure prompt treatment of pediatric MS patients. The presence of T1-weighted lesions in children, and particularly the greater frequency of such lesions, relative to adults, in the highly eloquent regions of the brainstem, raises concern regarding irreversible focal injury and the potential for future disability.
